# Sensitivity of fNIRS to cognitive state and load

**DOI:** 10.3389/fnhum.2014.00076

**Published:** 2014-02-20

**Authors:** Frank A. Fishburn, Megan E. Norr, Andrei V. Medvedev, Chandan J. Vaidya

**Affiliations:** ^1^Interdisciplinary Program in Neuroscience, Georgetown University Medical CenterWashington, DC, USA; ^2^Department of Psychology, Georgetown UniversityWashington, DC, USA; ^3^Center for Functional and Molecular Imaging, Georgetown University Medical CenterWashington, DC, USA; ^4^Children's National Medical Center, Children's Research InstituteWashington, DC, USA

**Keywords:** working memory, n-back, functional connectivity, resting state, fronto-parietal

## Abstract

Functional near-infrared spectroscopy (fNIRS) is an emerging low-cost noninvasive neuroimaging technique that measures cortical bloodflow. While fNIRS has gained interest as a potential alternative to fMRI for use with clinical and pediatric populations, it remains unclear whether fNIRS has the necessary sensitivity to serve as a replacement for fMRI. The present study set out to examine whether fNIRS has the sensitivity to detect linear changes in activation and functional connectivity in response to cognitive load, and functional connectivity changes when transitioning from a task-free resting state to a task. Sixteen young adult subjects were scanned with a continuous-wave fNIRS system during a 10-min resting-state scan followed by a letter n-back task with three load conditions. Five optical probes were placed over frontal and parietal cortices, covering bilateral dorsolateral PFC (dlPFC), bilateral ventrolateral PFC (vlPFC), frontopolar cortex (FP), and bilateral parietal cortex. Activation was found to scale linearly with working memory load in bilateral prefrontal cortex. Functional connectivity increased with increasing n-back loads for fronto-parietal, interhemispheric dlPFC, and local connections. Functional connectivity differed between the resting state scan and the n-back scan, with fronto-parietal connectivity greater during the n-back, and interhemispheric vlPFC connectivity greater during rest. These results demonstrate that fNIRS is sensitive to both cognitive load and state, suggesting that fNIRS is well-suited to explore the full complement of neuroimaging research questions and will serve as a viable alternative to fMRI.

## Introduction

Unprecedented technical advances in the past 20 years have made functional magnetic resonance imaging (fMRI) the primary neuroimaging modality for cognitive neuroscience. However, there are some notable drawbacks to fMRI that limit its utility in imaging young children and those with developmental disorders. First, head motion leads to substantial artifacts due to its relatively low temporal resolution and minimal constraint on head mobility in the scanning apparatus. Offline motion correction algorithms are effective for small movements, but larger movements necessitate subject exclusion, requiring oversampling as high as 30% in children with disorders such as Autism Spectrum Disorders (ASD), Attention Deficit Hyperactivity Disorder (ADHD), and Epilepsy (Yerys et al., 2009). In healthy young children (e.g., 4–6 years) who can comply with task instructions, the exclusion rate due to head motion was up to 40% (Yerys et al., 2009). Excessive head motion poses even more of a limitation for examining functional connectivity, the temporal co-activation of multiple brain regions, because even very small movements (e.g., <1 mm) introduce a systematic bias toward underestimating functional connectivity between distant regions (Power et al., 2012; Van Dijk et al., 2012). As the primary working hypothesis of some developmental disorders (e.g., ASD) is reduced long-distant functional connectivity, use of fMRI for those populations is particularly limiting. Second, the MR scanning environment is intimidating for many children. The enclosed nature of the scanning apparatus often produces feelings of claustrophobia, and the loud noise is fear-inducing for young children and autistic children with sensory hypersensitivity. Thus, despite its robust properties as a neuroimaging modality, fMRI is poorly suited for a large subset of pediatric and clinical populations. Thus, it is imperative to develop alternate neuroimaging modalities for investigating task-based and functional connectivity research questions.

Functional near-infrared spectroscopy (fNIRS) is an emerging non-invasive brain imaging modality for recording cortical hemodynamic activity. The method projects near-infrared light through the scalp and records optical density fluctuations resulting from metabolic changes within the brain. Similar to fMRI, cerebral blood flow is used as a proxy for neuronal activity. Both the spatial resolution and penetration depth of fNIRS are dependent upon the distances between light sources and detectors. The result is that the spatial resolution of fNIRS is on the order of 2.5–3 cm and is capable of imaging depths of 1–2 cm (McCormick et al., 1992), making it well-suited for imaging cortical regions. This technique is particularly resilient to contamination from head motion since the optodes are affixed to the head and thus move with the subject. The silent operation and unenclosed scanning environment make fNIRS more amenable to subjects that have sensory hypersensitivity or claustrophobia. These qualities of fNIRS make it particularly suitable for use with pediatric populations, including those with developmental disorders. While fNIRS has been used in functional neuroimaging for almost 30 years (Ferrari et al., 1985), it remains unclear whether fNIRS has the requisite sensitivity to serve as an alternative to fMRI. To that end, it is important that fNIRS be validated against cognitive phenomena with known neural bases. While fNIRS has potential for use with developmental and clinical populations, it is necessary to first validate its sensitivity for cognitive processes in healthy adults. Furthermore, in order for fNIRS to be considered as a viable alternative to fMRI for examining developmental disorders, it is vitally important that its sensitivity be validated on cognitive processes commonly affected in those disorders. The present study examines the sensitivity of fNIRS to changes in cognitive state (e.g., resting to task) and task load during working memory, a component process of higher cognition that is disrupted in numerous developmental and psychiatric disorders.

Working memory is a temporary buffer for active maintenance and manipulation of goal-relevant information that critically depends upon the integrity of prefrontal cortex and its connections with posterior brain regions (Miller and Cohen, 2001). fMRI studies have consistently shown activation within the dorsolateral prefrontal cortex (dlPFC) and posterior parietal cortex, with significant left-hemisphere lateralization in prefrontal cortex for verbal working memory tasks (for a meta-analysis, see Owen et al., 2005). More specifically, studies have shown that activation in left dlPFC scales linearly with working memory load (Braver et al., 1997; Jansma et al., 2000; Veltman et al., 2003), indicating load-dependent recruitment of dlPFC. Common manipulations of load in verbal working memory tasks involve linear increases in the size or temporal lag of to-be-remembered information. For example, on the n-back task, letters are presented serially with instructions to detect target letters that repeat, successively (low load, termed 1-back) or with 2 or 3 intervening trials (higher load, termed 2-back, and 3-back, respectively). Working memory capacity predicts higher cognitive ability indexed by general intelligence (Kane et al., 2005; Oberauer et al., 2005) and reasoning (Süß et al., 2002). It increases during development (Gathercole et al., 2004) and those age-related increases relate to frontal-parietal white-matter maturation (Nagy et al., 2004) and activation (Olesen et al., 2003). Working memory is impaired in several developmental disorders (Alloway et al., 2009) and its training improves higher cognition, such as reasoning (Jaeggi et al., 2008) and attention in ADHD (Klingberg et al., 2005). Further, training-related changes are reflected in frontal and parietal activation (Olesen et al., 2004). Thus, working memory is an optimal candidate for validation of fNIRS as it is crucial for higher cognition, sensitive to developmental pathology and intervention, and depends upon prefrontal and parietal cortices.

A number of fNIRS studies have examined the effect of varying working memory load on activation. The studies have generally found that higher working memory load tends to produce greater activation within dlPFC (Hoshi et al., 2003; Li et al., 2010; Ayaz et al., 2012; Molteni et al., 2012). Further, fNIRS has demonstrated sensitivity to group differences in activation during working memory, based on gender (Li et al., 2010), ADHD diagnosis (Ehlis et al., 2008), schizophrenia diagnosis (Lee et al., 2008), dopamine receptor genotype (Herrmann et al., 2007), and mild cognitive impairment in the elderly (Niu et al., 2013b). However, no fNIRS study has demonstrated dlPFC activation that scales linearly with working memory load. As the utility of fNIRS is contingent upon its robustness as an imaging modality, it is important to demonstrate that fNIRS is sensitive enough to detect the linear relationship between prefrontal activation and working memory load that has been documented with fMRI (Braver et al., 1997; Jansma et al., 2000; Veltman et al., 2003). Additionally, no study has examined functional connectivity during working memory with fNIRS. Thus, it is unknown whether fNIRS is capable of detecting working memory load-dependent changes in functional connectivity. Task-evoked functional connectivity measurement by fNIRS, is itself novel with only a handful of studies to date (Chaudhary et al., 2011; Medvedev et al., 2011; Hall et al., 2013).

It is presently not known whether fNIRS is sensitive to changes in cognitive state. Sensitivity to changes in cognitive state from drowsy to wakeful to cognitively engaged can be detected reliably with scalp-based electroencephalography (Schomer and Da Silva, 2010). Recent fMRI studies have shown how cortical functional networks change as subjects transition from resting/awake to cognitively engaged states (Gordon et al., 2012a,b). Specifically, functional connectivity was greater during working memory than rest between dlPFC and inferior parietal cortex (Gordon et al., 2012b). State-dependent changes are important to understand as they depend upon genetic factors (Gordon et al., 2012b) and can reflect consolidation associated with learning (Lewis et al., 2009). Further, task-free resting state, itself, is sensitive to individual variation in a variety of affective and behavioral traits (Vaidya and Gordon, 2013). Thus, demonstrating that fNIRS is sensitive to cognitive state is important to establish its versatility as a tool that is as suitable for the full complement of research questions as other neuroimaging modalities.

Previous investigations have found that resting-state networks can be detected with fNIRS (White et al., 2009; Lu et al., 2010; Mesquita et al., 2010; Zhang et al., 2010a,b), and are stable across testing sessions (Zhang et al., 2011). Resting-state networks have been shown to be segregated within different frequency bands (Sasai et al., 2011) and correlate with networks detected by simultaneous fMRI (Sasai et al., 2012). Graph theory approaches have also been successfully applied to resting-state fNIRS (Niu et al., 2012), demonstrating its sensitivity to the topological organization of resting-state networks and that these measurements are stable across testing sessions (Niu et al., 2013a). However, no studies have used fNIRS to investigate functional connectivity differences between the resting state and a cognitive task.

The present study addressed two questions: First, is fNIRS sensitive to load-dependent working memory changes in activation and functional connectivity in prefrontal-parietal regions? Second, is fNIRS sensitive to functional connectivity differences between working memory and a task-free resting state? Healthy young adult subjects were imaged during a 10-min task-free resting state followed immediately by a verbal n-back task, with three loads, 1-back, 2-back, and 3-back. We hypothesized that: (1) activation within prefrontal cortex would scale with n-back load, (2) fronto-parietal functional connectivity would scale with n-back load, and, (3) fronto-parietal functional connectivity would be greater during task than rest.

## Method

### Subjects

Sixteen Georgetown University undergraduates (6 male; 1 left-handed) ages 18–24 years (Mean ± *SD* = 20.3 ± 1.7) participated in the study for payment. Participants were not using psychotropic medication (e.g., stimulants, anti-depressants, anxiolytics) and had no history of neurological injury or disease, seizure disorder, or psychiatric diagnosis. All participants provided informed consent according to guidelines of the Georgetown University Institutional Review Board, which approved the protocol.

### Task procedure

fNIRS sessions consisted of a 10-min resting-state run in which the subjects were instructed to close their eyes and remain awake, followed by a 6.5 min n-back task. The sequence of rest and task was not counter-balanced due to previous research showing that task-induced changes in functional connectivity persist after task completion (Evers et al., 2012; Gordon et al., 2012a; Harmelech et al., 2013). During the n-back task, participants were presented with a series of single consonant letters and instructed to press a button with their dominant hand when the presented letter was the same as the one presented *n* letters ago. Subjects were tested on three blocks of each of the three load conditions: 1-back, 2-back, and 3-back. The load condition order was pseudorandomized using a modified Latin square. Each block consisted of 9 trials, each lasting 3000 ms, with the letter exposed for 500 ms followed by a lag of 2500 ms. Each 27-s block was followed by a 14-s interval of fixation to allow the hemodynamic response to return to baseline. Subjects practiced the n-back task prior to the scanning session.

### Imaging procedure

**Figure 1:**
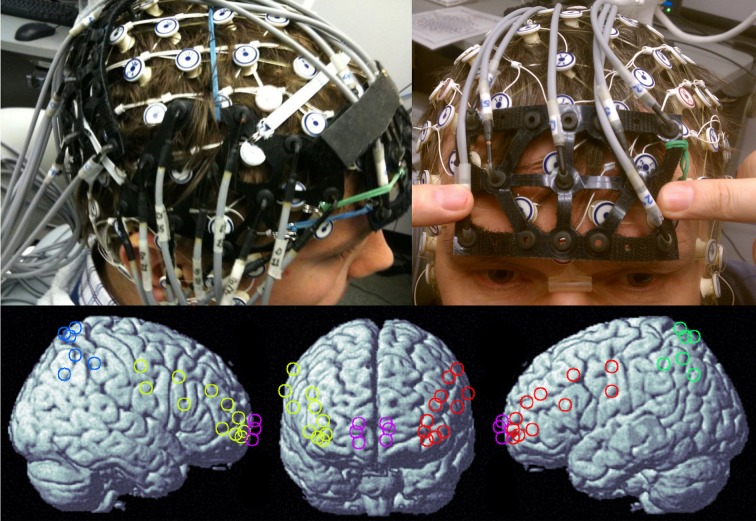
**NIRS probe configuration**. Upper left panel shows the right parietal and right frontal probes and the upper right panel shows the medial frontal probe. Bottom panel is a rendering of estimated channel positions on a template brain. Colors indicate the channel sets used for interpolation.

Optical signals were recorded on a two-wavelength (690 and 830 nm) continuous-wave CW5 imaging system (TechEn, Inc., Milford, MA). Data were collected from detectors in parallel at a sampling rate of 41.7 kHz, with each laser modulated at a different frequency to allow subsequent offline demodulation and separation of source-detector pairs (i.e., channels). The 40 optical channels were comprised of 12 sources and 29 detectors, arranged into 5 probes, covering bilateral parietal cortex, bilateral prefrontal cortex, and frontal pole. Participants were fitted with a 128-channel HydroCel EEG cap (Electrical Geodesics, Inc., Eugene, OR) prior to probe placement. The cap provided a consistent frame of reference for positioning optical probes. Optode coordinates (provided in Table 1) in 10–20 reference space were estimated by triangulation with the three nearest EEG electrodes, using the electrode coordinates provided by the manufacturer. The NFRI software package (Singh et al., 2005) was then used to generate interpolation kernels for projection of channel data onto the brain surface, with interpolation only taking place between channels on the same probe (Figure 1).

**Table 1:** **Optode positions computed from distances to neighboring EEG electrodes**.

**Region**	**Optode**	***X***	***Y***	***Z***
Left dl/vlPFC	S01	−5.5848916	5.9299802	0.8992566
Left dl/vlPFC	S02	−6.5386068	3.5896627	2.5317016
Left dl/vlPFC	S03	−6.8767829	1.4968671	3.1051698
Right dl/vlPFC	S04	5.5848916	5.9299802	0.8992566
Right dl/vlPFC	S05	6.5386068	3.5896627	2.5317016
Right dl/vlPFC	S06	6.8767829	1.4968671	3.1051698
Left parietal	S07	−3.2937507	−3.5253026	7.6092289
Left parietal	S08	−6.8859022	−2.4269973	3.9041052
Right parietal	S09	3.2937507	−3.5253026	7.6092289
Right parietal	S10	6.8859022	−2.4269973	3.9041052
Frontal pole	S11	1.9829266	9.1819183	0.0705624
Frontal pole	S12	−1.9829266	9.1819183	0.0705624
Left dlPFC	D01	−4.0697039	7.8642451	0.0136511
Left vlPFC	D02	−5.0047896	6.7598403	−1.9661292
Left dlPFC	D03	−4.7733352	6.5438124	2.9521678
Left vlPFC	D04	−6.2841462	4.3467260	−1.6610823
Left dlPFC	D05	−5.2295352	5.2107974	3.9431675
Left vlPFC	D06	−7.0997050	1.3796777	−0.0546826
Left dlPFC	D07	−5.9805701	2.8239378	4.6847051
Right dlPFC	D08	4.0697039	7.8642451	0.0136511
Right vlPFC	D09	5.0047896	6.7598403	−1.9661292
Right dlPFC	D10	4.7733352	6.5438124	2.9521678
Right vlPFC	D11	6.2841462	4.3467260	−1.6610823
Right dlPFC	D12	5.2295352	5.2107974	3.9431675
Right vlPFC	D13	7.0997050	1.3796777	−0.0546826
Right dlPFC	D14	5.9805701	2.8239378	4.6847051
Left parietal	D15	−2.0340789	−1.3669953	8.4747830
Left parietal	D16	−2.8070659	−5.1200481	7.0390415
Left parietal	D17	−5.5694535	−2.8568375	6.1385888
Left parietal	D18	3.6944529	7.0014432	−2.6781789
Left parietal	D19	−7.1484855	−0.3844012	3.3718455
Right parietal	D20	−6.4584192	−4.8154434	0.8565472
Right parietal	D21	2.8070659	−5.1200481	7.0390415
Right parietal	D22	2.0340789	−1.3669953	8.4747830
Right parietal	D23	5.5694535	−2.8568375	6.1385888
Right parietal	D24	3.3304629	8.0217702	2.7577466
Frontal pole	D25	6.4584192	−4.8154434	0.8565472
Frontal pole	D26	7.1484855	−0.3844012	3.3718455
Frontal pole	D27	0.0000000	9.1241807	0.6243727
Frontal pole	D28	−3.6944529	7.0014432	−2.6781789
Frontal pole	D29	−3.3304629	8.0217702	2.7577466

Source optodes are denoted with S, and detector optodes are denoted with D. Source optodes in PFC were located along the boundary between dlPFC and vlPFC, with detector optodes above and below giving rise to dlPFC and vlPFC channels, respectively.

### Behavioral data analysis

Behavioral data were lost for 2 subjects due to computer malfunction. Subject accuracy was computed for each load condition by taking the mean percentage of correct trials. Reaction time was computed by taking the mean across correct trials within each load condition. Repeated-measures ANOVAs and paired *t*-tests were performed for both accuracy and reaction time.

### fNIRs preprocessing

Data were low-pass filtered with a high-order (400) FIR filter at 0.8 Hz and downsampled to 20 Hz. Raw optical density signals were converted to hemoglobin concentration changes using the modified Beer–Lambert law (Cope and Delpy, 1988) with the HOMer software package (Huppert et al., 2009). The oxy-Hb signal has previously been shown to correlate with blood flow better than the deoxygenated signal (Hoshi et al., 2001), thus interpretations focus on the oxygenated signal. Results for deoxygenated data are provided in the supplementary materials.

### fNIRs analysis—activation

Channel timecourses were modeled with a general linear model (GLM) in NIRS-SPM (Ye et al., 2009). Regressors were generated by convolving the weighted task boxcar function with the canonical hemodynamic response function provided by SPM8 (Friston et al., 1994). Data were corrected for low frequency drift by detrending using the wavelet-MDL algorithm (Jang et al., 2009) and corrected for serial correlations, such as those produced by physiological noise sources, using the HRF precoloring method (Worsley and Friston, 1995) implemented within NIRS-SPM (Ye et al., 2009). In order to separate the effects of load-dependent and load-independent activation, two regressors were generated: (1) a load-independent regressor in which all n-back blocks were weighted equally, and (2) a load-dependent regressor in which each n-back block was weighted by its load (i.e., 1, 2, or 3). Channel-wise beta values were compared across subjects for outliers. Subjects that had two or more adjacent channels with beta values over 3 standard deviations from the group mean were excluded from further analysis. Three subjects were excluded in this manner, thus reported results are from *N* = 13. The channel-wise beta values from the remaining subjects were then interpolated into voxel space. T-contrasts were then used to generate statistical parametric maps of activation for each regressor. A *p-value* correction was applied to control the family-wise error rate using the Lipschitz-Killing curvature-based Euler characteristic (EC) approach (Li et al., 2012). Activation maps were thresholded at a corrected threshold of *p* < 0.05.

### fNIRs analysis—functional connectivity

As fMRI studies have primarily found functional resting-state networks in the 0.01–0.10 Hz frequency range, both the resting-state data and n-back data were filtered around this range. This step also prevented high-frequency physiological artifacts from biasing the results. To this end, a band-pass Fourier filter was applied using the publicly available iFilter script for Matlab (Filter parameters: center = 0.035 Hz, width = 0.04 Hz, shape = 10; corresponding to a pass-band of approximately 0.009–0.09 Hz).

#### Load-dependent

For each n-back load, the individual block timecourses were concatenated and the Pearson correlation coefficient was computed between all channel-pairs. A Fisher's r-to-Z transformation was then applied to normalize the variance of the correlation values. For each channel-pair, the transformed correlation values were regressed against the corresponding n-back load. The t-statistic of the estimated beta value (i.e., the beta value divided by its standard error) was used in a one-sample *t*-test across subjects. Channels were grouped into 7 anatomical regions: left/right parietal (P), left/right ventrolateral PFC (vlPFC), left/right dorsolateral PFC (dlPFC), and frontal pole (FP). For each region-pair, a one-sample *t*-test was performed on the channel-wise *t*-statistic against the null hypothesis that the mean channel-wise *t*-statistic was less than the corresponding critical value at *p* < 0.05. A Bonferroni correction was applied to the region-wise *p*-values to control for multiple comparisons. The final significance threshold was set at *p* < 0.05.

#### State-dependent

To determine the contribution of cognitive state to functional connectivity, the resting-state and n-back scans were compared. Beginning and ending sections of the resting-state scan were removed to match the duration of the n-back task. In order to mitigate the influence of activation on the state-wise functional connectivity comparison, activation was regressed from the n-back fNIRS data. A GLM was created in NIRS-SPM with each load-level as a separate regressor. This GLM was necessary in order to remove non-linear load effects that would not be captured by the linear GLM used in the activation analyses. The residuals from the estimation were then used in the connectivity analyses. The results from the connectivity analyses without this regression step are provided in Supplementary Figures 4, 5. Regression of activation was not applied in the load-dependent functional connectivity analysis, as different n-back loads are directly comparable and make an interpretable contribution to load-dependent changes in functional connectivity. The Pearson correlation was computed between all pairs of channels and Fisher's r-to-Z transformation was applied. For each channel-pair, a paired *t*-test (n-back > rest) was computed across subjects. For each region-pair, a one-sample *t*-test was performed on the channel-wise *t*-statistic. A Bonferroni correction was applied to the region-wise *p*-values to control for multiple comparisons. The *p*-value threshold was set at *p* < 0.05.

## Results

### Task performance

**Figure 2:**
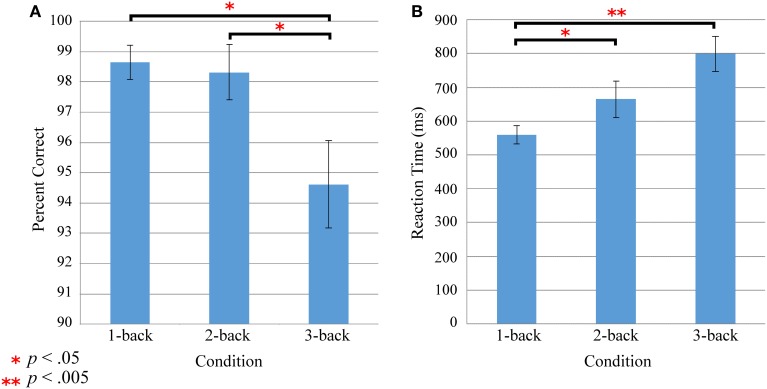
**Effect of n-back load on accuracy (A) and reaction time (B)**. The 3-back load condition had significantly lower accuracy than 1-back and 2-back conditions **(A)**. Reaction times for the 1-back condition were significantly faster than 2-back and 3-back **(B)**.

One-Way repeated-measures ANOVA with Greenhouse-Geisser correction revealed a main effect of load on accuracy [*F*_(1.768, 17.677)_ = 4.043, *p* < 0.05]. Paired *t*-tests indicated that mean accuracy for 1-back (98.7 ± 1.9%) and 2-back (98.3 ± 3.0%) were near ceiling and did not differ (Figure 2A). Accuracy for the 3-back (94.6 ± 4.8%) condition was significantly lower than the 1-back [*t*_(10)_ = 2.292, *p* < 0.05] and the 2-back [*t*_(10)_ = 2.236, *p* < 0.05].

One-Way repeated-measures ANOVA with Greenhouse-Geisser correction revealed a main effect of load on reaction time [*F*_(1.272, 12.722)_ = 7.697, *p* < 0.05]. Paired *t*-tests indicated that 1-back (559 ± 106 ms) was performed faster than the 2-back (665 ± 218 ms), *t*_(10)_ = 2.423, *p* < 0.05, and the 3-back (800 ± 207 ms), *t*_(10)_ = 4.513, *p* < 0.005, while the 2-back and 3-back did not differ (Figure 2B).

### Load-dependent activation

**Figure 3:**
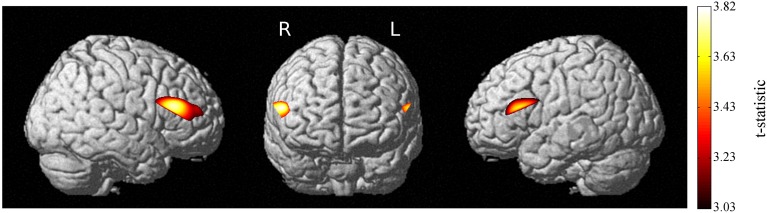
**Increases in activation with increasing working memory load**. Load-dependent activation is seen in bilateral prefrontal cortex. *p* < 0.05, EC-corrected.

Load-dependent oxy-Hb activation was significant in bilateral dlPFC (Figure 3), showing that the engagement of these regions increased linearly as working memory load increased from 1-back to 3-back trials on the verbal n-back task. Activation for the load-independent regressor, in which all blocks were weighted equally, did not survive correction.

### Load-dependent functional connectivity

**Figure 4:**
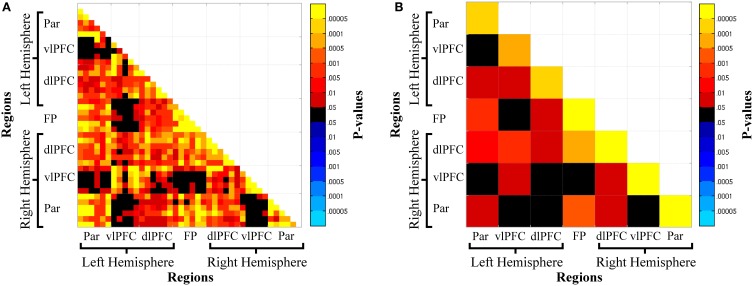
**Channel-wise (A) and region-wise (B) load-dependent functional connectivity matrices**. Warm colors denote a load-dependent increase in functional connectivity, while cool colors denote load-dependent decrease in functional connectivity. Abbreviations: Par, Parietal; vlPFC, ventrolateral prefrontal cortex; dlPFC, dorsolateral prefrontal cortex; FP, frontal pole.

Oxy-Hb functional connectivity increased with increasing n-back loads: (1) between hemispheres for parietal cortex, L-Par—R-Par, and prefrontal cortex, L-vlPFC—R-vlPFC, L-dlPFC—R-dlPFC, L-vlPFC—R-dlPFC; (2) between frontal and parietal regions within the left (L-Par—L-dlPFC) and right (R-Par—R-dlPFC) hemispheres, across hemispheres (L-Par—R-dlPFC), and between parietal and frontopolar cortex (R-Par—FP, L-Par—FP); (3) between adjacent regions in frontal cortex, L-vlPFC—L-dlPFC, R-vlPFC—R-dlPFC, FP—L-dlPFC, FP—R-dlPFC; and (4) within all regions: L-Par, R-Par, L-vlPFC, R-vlPFC, L-dlPFC, R-dlPFC, FP (Figures 4A,B).

### State-dependent functional connectivity

**Figure 5:**
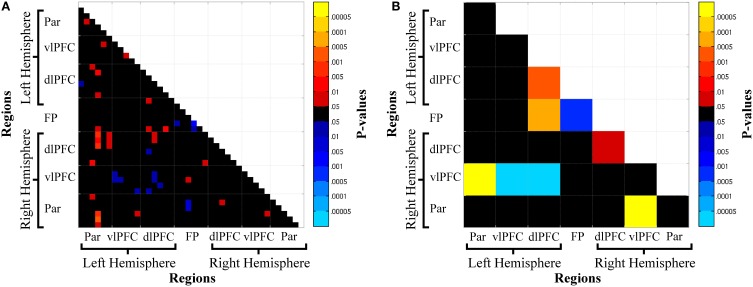
**Channel-wise (A) and region-wise (B) state-dependent functional connectivity matrices**. Warm colors denote connections where functional connectivity was greater during the n-back than at rest, while cool colors denote greater connectivity at rest. Abbreviations: Par, Parietal; vlPFC, ventrolateral prefrontal cortex; dlPFC, dorsolateral prefrontal cortex; FP, frontal pole.

Oxy-Hb functional connectivity increased from the resting-state run to n-back task: (1) between frontal and parietal regions, L-Par—R-vlPFC, R-Par—R-vlPFC; (2) between adjacent frontal regions, L-dlPFC—FP, R-dlPFC—FP; and (3) within bilateral dlPFC, R-dlPFC, L-dlPFC (Figure 5). In contrast, functional connectivity decreased from the resting-state run to the n-back task: (1) between homologous frontal regions: L-vlPFC—R-vlPFC, L-dlPFC—R-vlPFC. Thus, task-engagement resulted in an increase of functional connectivity between right vlPFC and bilateral parietal cortex, within bilateral dlPFC, and between bilateral dlPFC and FP. Functional connectivity decreased from rest to task between right vlPFC and left PFC (vlPFC and dlPFC).

## Discussion

The present study addressed two questions: (1) whether fNIRS is sensitive to load-dependent working memory changes in activation and functional connectivity in prefrontal-parietal regions, and (2) whether fNIRS is sensitive to functional connectivity differences between a working memory task and a task-free resting state. Activation was found to increase linearly with working memory load in bilateral PFC. Functional connectivity increased with working memory load between frontal and parietal regions, between hemispheres for homologous frontal and parietal regions, and locally (i.e., within regions and between adjacent regions). Change in cognitive state, from resting to working memory, changed functional connectivity such that it increased in fronto-parietal connections but decreased in inter-hemispheric frontal connections. These results collectively demonstrate that fNIRS detected functional neural changes associated with modulation of cognitive load and state in frontal and parietal cortices.

Working memory load-dependent activation increased linearly in bilateral dorsolateral prefrontal cortex, with stronger activation in the left hemisphere. This load-dependent prefrontal activation is consistent with previous fMRI findings (Braver et al., 1997; Jansma et al., 2000; Veltman et al., 2003). Braver et al. (1997) used a verbal n-back with loads of 0-, 1-, 2-, and 3-back and found load-dependent activation in bilateral dlPFC and left vlPFC. Jansma et al. (2000) used a spatial n-back task with loads 0-, 1-, 2-, and 3-back and found load-dependent activation in bilateral dlPFC and parietal cortex. Veltman et al. (2003) used a verbal n-back with loads of 0-, 1-, 2-, and 3-back and found load-dependent activation in bilateral dlPFC, left vlPFC, and left parietal cortex. Although found in fMRI studies, parietal activation did not survive corrected threshold in the present study. It has been shown that parietal regions have a longer scalp-to-brain distance than frontal regions and that this increased distance results in lower signal-to-noise ratio, as measured by correlation with simultaneous fMRI (Cui et al., 2011). Thus, the increased distance between scalp and brain may have impeded detection of activation in parietal cortex. Most previous fNIRS studies using a verbal n-back task examined load effects in a pairwise manner: subtracting the mean signal change of 1-, 2-, and 3-back each from 0-back (Hoshi et al., 2003), comparing mean signal change of 1-, 2-, and 3-back in an ANOVA (Li et al., 2010), and using a GLM to compare 1-, 2-, 3-back each with 0-back and baseline (Molteni et al., 2012). Ayaz et al. (2012) used a repeated-measures ANOVA to find a main effect of load on activation in left PFC, though *post-hoc* analyses showed only that 3-back had greater activation that 0- and 1-back. The present study is the first to use fNIRS to test for linear increases of activation spanning multiple working memory loads. As optode coverage was limited to prefrontal, parietal, and frontal pole, it is unknown whether other regions such as visual or temporal cortices were also sensitive to load-related increase. Establishing the sensitivity of fNIRS to prefrontal load-dependent modulation provides a useful tool for detecting both immature and disordered functional anatomy underlying higher order cognition. Working memory capacity predicts a variety of higher cognitive functions including reading ability (Swanson and Jerman, 2007), reasoning and problem solving (Süß et al., 2002), and general intellectual function as indexed by IQ (Kane et al., 2005; Oberauer et al., 2005). Prefrontal response to working memory demands depends upon dopaminergic activity (Aalto et al., 2005; McNab et al., 2009). Therefore, a load-dependent working-memory fNIRS probe is likely to be a useful tool in detecting disturbances in prefrontal functioning supporting higher cognition.

Functional connectivity was found to increase with working memory load between frontal and parietal regions, between hemispheres for homologous frontal and parietal regions, and locally (i.e., within regions and between adjacent regions). This finding supports the view that working memory is supported in a load-dependent manner by communication between prefrontal and parietal cortices, as well as between hemispheres. Notably, fronto-parietal functional connectivity increased with load for dlPFC, but not vlPFC. These findings are in accord with previous fMRI research showing load-dependent functional connectivity between contralateral and ipsilateral prefrontal and parietal regions, with stronger parietal connectivity with dlPFC than with vlPFC (Narayanan et al., 2005). This is the first demonstration of fNIRS sensitivity to functional connectivity changes related to working memory load, establishing the utility of fNIRS for probing task-evoked functional connectivity. It is of particular importance that fNIRS be shown to have sensitivity to load-related changes in functional connectivity, as this may allow functional connectivity to serve as a proxy for structural connectivity in those who cannot be imaged with traditional methods. Structural brain connectivity is typically assessed using Diffusion Tensor Imaging (DTI), an MRI technique that estimates the integrity of white-matter tracts. The reliance upon MRI precludes its use with a large subset of the developmental and clinical populations. This is particularly troublesome for developmental disorders such as ASD, which are associated with disruptions in connectivity (Courchesne and Pierce, 2005; Just et al., 2012). While fNIRS does not provide structural information directly, functional connectivity may provide indirect structural information. Functional connectivity depends, at least in part, upon the quality of structural connections between regions, and previous fMRI work has shown that functional connectivity predicts white matter integrity (Gordon et al., 2011). By measuring the relative changes of functional connectivity across varying task loads, the strength of the underlying structural connections may be estimated. In this way, fNIRS could prove to be a valuable tool for assessment of brain connectivity in populations that cannot currently be reached by DTI. However, fNIRS must first be capable of detecting connectivity differences across workloads. The present demonstration that fNIRS is sensitive to changes in functional connectivity resulting from working memory load provides further support for the potential of fNIRS in this domain.

The flexible engagement and disengagement of cognitive resources for serving current goals is the hallmark of mature cognition. Set-shifting, the ability to switch between response sets, is a form of cognitive flexibility that continues developing through early adulthood (Kalkut et al., 2009). Furthermore, set-shifting is impaired in developmental disorders of executive function such as ASD (Maes et al., 2011). Therefore, this form of flexibility is a vulnerable component of executive function. We reasoned that the simplest case of such flexibility is the transition from a resting to a task-performing state—the resting state can be thought of as one of unconstrained attention (as subjects are told to not think of anything in particular but to stay awake) when contrasted with n-back performance where attention has to be constrained to evaluating letters for n-back targets. fMRI studies show that fronto-parietal functional connectivity becomes stronger as subjects transition to a task from being at rest, and most importantly, individual variation in the magnitude of state-related functional connectivity changes predicted attentional function in everyday life (Gordon et al., 2012a,b). Here, we found that increased fronto-parietal functional connectivity was accompanied by reduced interhemispheric frontal connectivity, as subjects transitioned from rest to the task. A task-related decrease in functional connectivity between homologous prefrontal regions may be the result of a task-related increase of functional lateralization. Activation is commonly found to be stronger in the left hemisphere in verbal working memory paradigms (Owen et al., 2005). It stands to reason that an unconstrained resting state may have greater inter-hemispheric connectivity than a task that places demands on functions that are strongly lateralized. These changes were not driven by load-related variability in functional connectivity, because loads were regressed out. In children with connectivity abnormalities, such as those with ASD, state-related changes in functional connectivity suggested lack of engagement of task-selective circuitry and predicted variability on inattention symptoms among school-aged children (You et al., 2013). Thus, the availability of an accessible imaging modality that is sensitive to state-related changes in functional communication will be useful for investigation of both normal and disordered cognition. This demonstration of the sensitivity of fNIRS to cognitive state is an important step toward measuring cognitive flexibility.

The present findings need to be considered in the context of the following methodological issues: First, while fNIRS has excellent temporal resolution and resilience to artifacts arising from head motion, the spatial resolution is inferior to fMRI. Although fitted EEG caps were used to position NIRS probes in reference to standard 10–20 coordinates, the location of channels with respect to underlying brain regions could not be independently verified. These factors make precise localization of fNIRS signals particularly difficult. However, the spatial resolution of fNIRS is low enough (2–3 cm) that these relatively small imprecisions should have only a minimal impact on the results. Second, while the medial frontal channels are likely to overlap with frontopolar cortex, there is some difficulty in interpretation due to greater depth of cortex as it approaches the medial longitudinal fissure. Thus, medial frontal channels may have covered regions where cortex was further from the scalp, potentially weakening the signal relative to lateral prefrontal cortices. Third, while the deoxygenated hemoglobin signal tended to show similar patterns of activation (Supplementary Figure 1) and functional connectivity (Supplementary Figures 2, 3) to the oxygenated signal, the patterns were not identical. Given that task-evoked influx of oxygenated hemoglobin far outpaces oxygen metabolism, it is not surprising that the oxygenated hemoglobin signal is more robust than the deoxygenated signal. Further, deoxygenated hemoglobin signal tends to have lower signal-to-noise ratio than oxygenated, due in part to lesser tissue penetration of the short-wavelength light associated with deoxygenated signal. Fourth, task performance did not scale linearly with n-back load. This is not surprising as performance is limited by the sensitivity of the task to measure differences and is subject to ceiling and floor effects. In addition, subjects are often able to accommodate increased workloads without a significant change in performance simply by increasing effort. In contrast, brain activation is closely linked to effort and is thus expected to be more sensitive to changes in workload than behavior. This is evidenced by studies showing group differences in brain activation where performance does not differ (Matsuo et al., 2006; Herrmann et al., 2007). Therefore it is not necessary for task performance to scale with working memory load. Despite the limitations, our findings show some useful attributes of prefrontal-parietal functioning, specifically sensitivity to working memory load and cognitive state. This sensitivity makes fNIRS a useful imaging modality for a large segment of children and adults who cannot be reached by fMRI. As this field matures, some consensus will emerge regarding data processing steps and parameter choices that ought to make it possible to compare results across studies more reliably.

In sum, this study demonstrates the utility of fNIRS for detection of activation and functional connectivity related to cognitive load and state. These findings are particularly important as they provide a basis for the use of fNIRS as an alternative to fMRI in studies of executive function, particularly in pediatric and clinical populations that are not amenable to fMRI. Working memory is an important domain in this regard, as it develops over the course of childhood and adolescence and is subverted in developmental disorders. This study is the first to show that fNIRS has the requisite sensitivity to detect activation and functional connectivity that increase linearly with increasing working memory load, and one of the first to demonstrate that fNIRS can reliably detect differences related to cognitive state (e.g., rest versus task). In order for fNIRS to be adopted for widespread use, it is vital to first demonstrate its sensitivity to activation and functional connectivity during cognitive processes of interest.

### Conflict of interest statement

The authors declare that the research was conducted in the absence of any commercial or financial relationships that could be construed as a potential conflict of interest.

## Supplementary material

**Load-dependent Deoxy-Hb activation**

Load-dependent activation for the deoxy-Hb signal did not survive correction, but was patterned similarly to oxy-Hb with bilateral frontal activation (Supplementary Figure 1). Load-independent activation was not observed.

**Load-dependent Deoxy-Hb functional connectivity**

Load-dependent deoxy-Hb functional connectivity reached corrected threshold in frontal cortex only, increasing with greater n-back loads: for (1) fronto-parietal connections (FP-lP, FP-rP); (2) frontal interhemispheric connections (lvlPFC-rvlPFC); (3) parieto-parietal connections (lP-rP); and local connections (rvlPFC-rdlPFC, lP, lvlPFC, ldlPFC, FP, rdlPFC, rvlPFC, rP; Supplementary Figure 2).

**State-dependent Deoxy-Hb functional connectivity**

Deoxy-Hb functional connectivity was greater during the n-back run than the resting state run between (Supplementary Figure 3): (1) frontal and parietal regions (FP-lP, rdlPFC-lP, rvlPFC-lP, rdlPFC-rP); (2) between parietal regions (lP-rP); and within regions (rP, lP, rdlPFC). Functional connectivity was greater for resting-state than n-back: (1) for all significant connections with left frontal regions (lvlPFC-ldlPFC, lvlPFC-FP, ldlPFC-FP, ldlPFC-rdlPFC, lvlPFC-rvlPFC, ldlPFC-rvlPFC); (2) between frontal and parietal regions: rP-lvlPFC; and (3) within frontal regions (ldlPFC, rvlPFC, FP).

**State-dependent functional connectivity without task regression**

Oxy-Hb signal showed task-related functional connectivity that was much more prominent than in the task-regression case for: (1) fronto-parietal connections (lvlPFC-lP, ldlPFC-lP, rdlPFC-lP, lvlPFC-rP, ldlPFC-rP, FP-rP, rdlPFC-rP); (2) between hemispheres for parietal (lP-rP); and (3) between bilateral dlPFC and FP (ldlPFC-rdlPFC, ldlPFC-FP, rdlPFC-FP). Similarly, the deoxygenated signal showed greater fronto-parietal (lvlPFC-lP, ldlPFC-lP, FP-lP, rdlPFC-lP, FP-rP, rdlPFC-rP), parieto-parietal (lP-rP, lP-lP, rP-rP), and fronto-frontal (vlPFC-FP, dlPFC-FP) task-related functional connectivity (Supplementary Figure 5) relative to the task-regressed results. The increase in task-related functional connectivity is interpreted as the result of bias from the presence of task-related activation.

Supplementary Figure 1 **Load-dependent activation from the deoxygenated hemoglobin signal shared a similar pattern with the oxygenated signal, but did not reach significance**. *p* < 0.05, uncorrected.

Supplementary Figure 2 **Channel-wise (A) and region-wise (B) load-dependent functional connectivity matrices for the deoxygenated signal**. Warm colors denote a load-dependent increase in functional connectivity, while cool colors denote load-dependent decrease in functional connectivity. Abbreviations: Par, Parietal; vlPFC, ventrolateral prefrontal cortex; dlPFC, dorsolateral prefrontal cortex; FP, frontal pole.

Supplementary Figure 3 **Channel-wise (A) and region-wise (B) state-dependent functional connectivity matrices from deoxygenated hemoglobin signal**. Warm colors denote connections where functional connectivity was greater during the n-back than at rest, while cool colors denote greater connectivity at rest. Abbreviations: Par, Parietal; vlPFC, ventrolateral prefrontal cortex; dlPFC, dorsolateral prefrontal cortex; FP, frontal pole.

Supplementary Figure 4 **Channel-wise (A) and region-wise (B) state-dependent functional connectivity matrices without performing regression on the task data (oxygenated hemoglobin signal)**. Warm colors denote connections where functional connectivity was greater during the n-back than at rest, while cool colors denote greater connectivity at rest. Abbreviations: Par, Parietal; vlPFC, ventrolateral prefrontal cortex; dlPFC, dorsolateral prefrontal cortex; FP, frontal pole.

Supplementary Figure 5 **Channel-wise (A) and region-wise (B) state-dependent functional connectivity matrices without performing regression on the task data (deoxygenated hemoglobin signal)**. Warm colors denote connections where functional connectivity was greater during the n-back than at rest, while cool colors denote greater connectivity at rest. Abbreviations: Par, Parietal; vlPFC, ventrolateral prefrontal cortex; dlPFC, dorsolateral prefrontal cortex; FP, frontal pole.
