# Supplementary figures and images for: Sensitivity of fNIRS to cognitive state and load
Source: Front Hum Neurosci. 2014 Feb 20;8:76. doi: 10.3389/fnhum.2014.00076 (PMC3930096; doi:10.3389/fnhum.2014.00076)

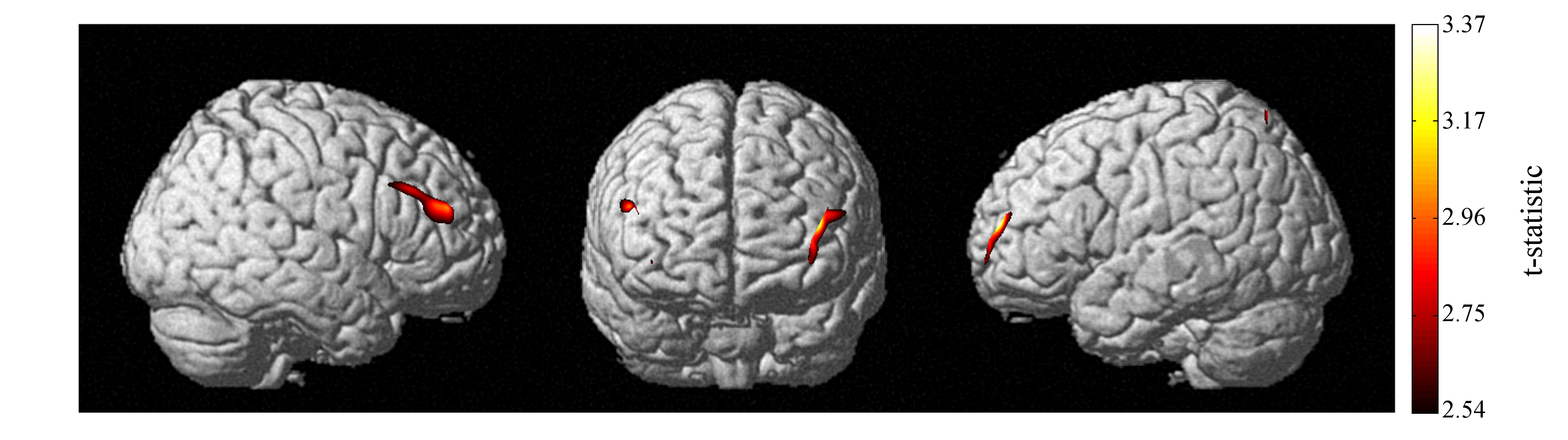

Supplement: Supplementary Figure 1 — Load-dependent activation from the deoxygenated hemoglobin signal shared a similar pattern with the oxygenated signal, but did not reach significance. p < 0.05, uncorrected. [file Data_Sheet_1.ZIP › Supplementary Figures/Figure S1.TIF]

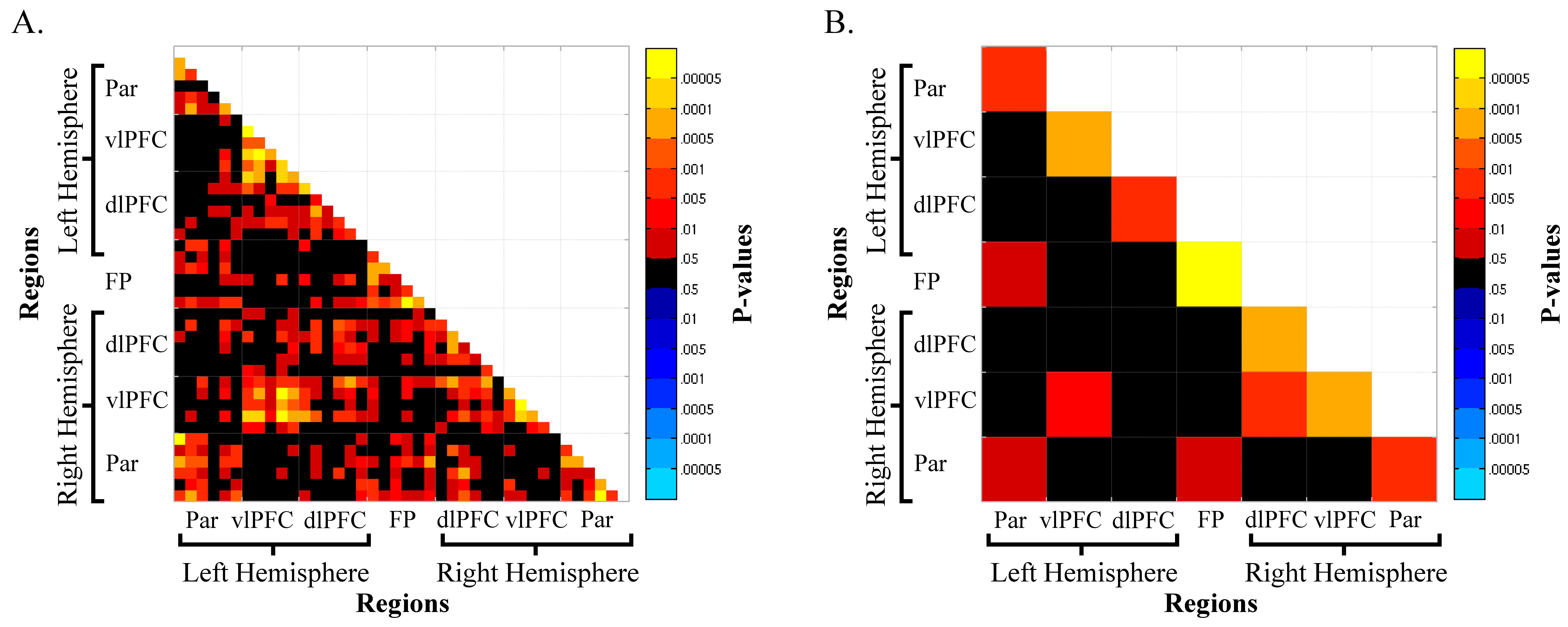

Supplement: Supplementary Figure 1 — Load-dependent activation from the deoxygenated hemoglobin signal shared a similar pattern with the oxygenated signal, but did not reach significance. p < 0.05, uncorrected. [file Data_Sheet_1.ZIP › Supplementary Figures/Figure S2.TIF]

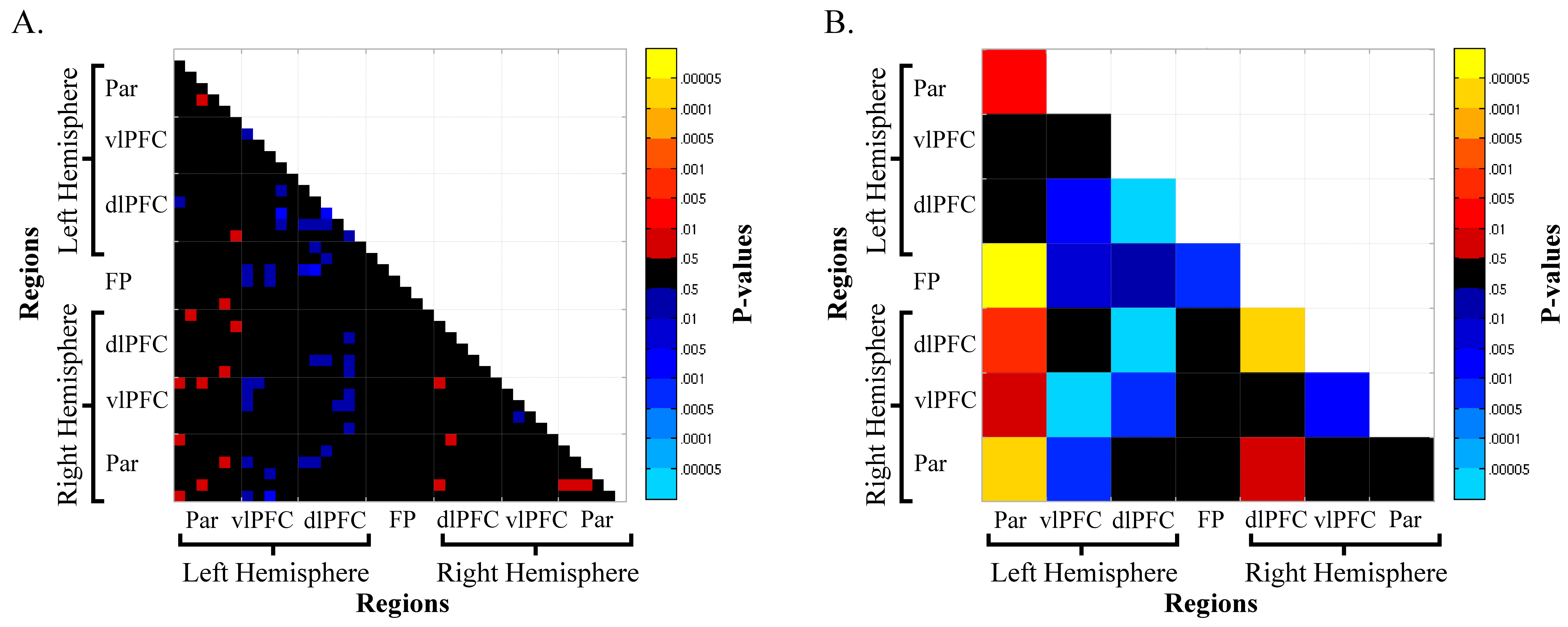

Supplement: Supplementary Figure 1 — Load-dependent activation from the deoxygenated hemoglobin signal shared a similar pattern with the oxygenated signal, but did not reach significance. p < 0.05, uncorrected. [file Data_Sheet_1.ZIP › Supplementary Figures/Figure S3.TIF]

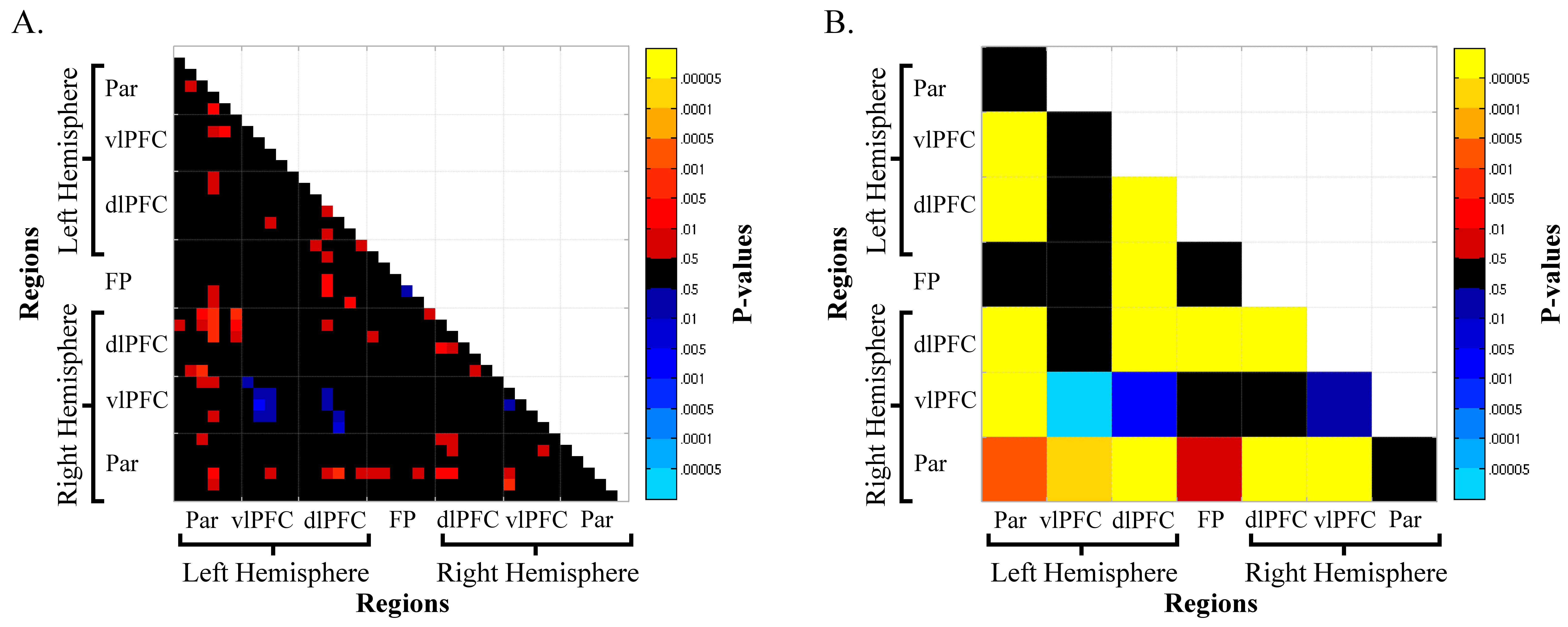

Supplement: Supplementary Figure 1 — Load-dependent activation from the deoxygenated hemoglobin signal shared a similar pattern with the oxygenated signal, but did not reach significance. p < 0.05, uncorrected. [file Data_Sheet_1.ZIP › Supplementary Figures/Figure S4.TIF]

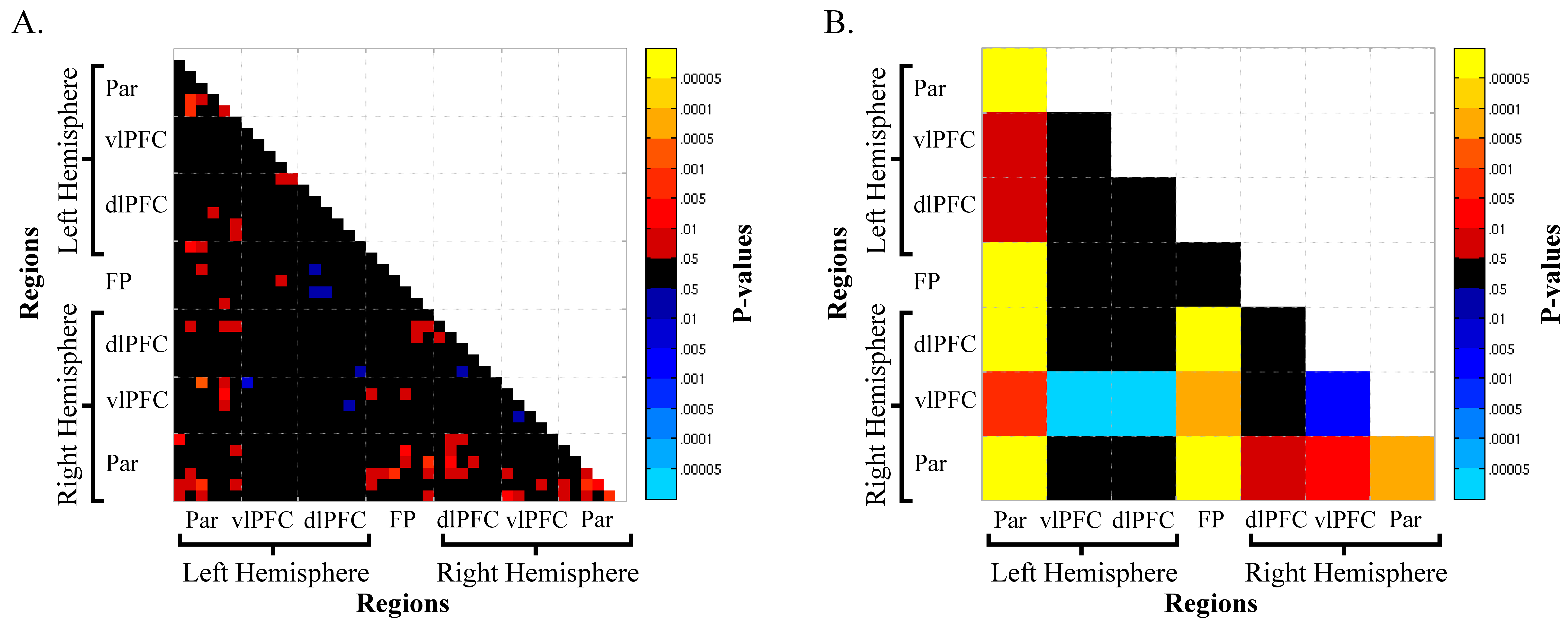

Supplement: Supplementary Figure 1 — Load-dependent activation from the deoxygenated hemoglobin signal shared a similar pattern with the oxygenated signal, but did not reach significance. p < 0.05, uncorrected. [file Data_Sheet_1.ZIP › Supplementary Figures/Figure S5.TIF]
